# Grid diagrams as tools to investigate knot spaces and topoisomerase-mediated simplification of DNA topology

**DOI:** 10.1126/sciadv.aay1458

**Published:** 2020-02-26

**Authors:** Agnese Barbensi, Daniele Celoria, Heather A. Harrington, Andrzej Stasiak, Dorothy Buck

**Affiliations:** 1 Mathematical Institute, University of Oxford, Oxford, UK.; 2 Center for Integrative Genomics, University of Lausanne, Lausanne, Switzerland.; 3 SIB Swiss Institute of Bioinformatics, Lausanne, Switzerland.; 4 Department of Mathematical Sciences, University of Bath, Bath, UK.; 5 Department of Mathematics/Biology, Trinity College of Arts and Sciences, Duke University, Durham, NC, USA.

## Abstract

Grid diagrams with their relatively simple mathematical formalism provide a convenient way to generate and model projections of various knots. It has been an open question whether these 2D diagrams can be used to model a complex 3D process such as the topoisomerase-mediated preferential unknotting of DNA molecules. We model here topoisomerase-mediated passages of double-stranded DNA segments through each other using the formalism of grid diagrams. We show that this grid diagram–based modeling approach captures the essence of the preferential unknotting mechanism, based on topoisomerase selectivity of hooked DNA juxtapositions as the sites of intersegmental passages. We show that the grid diagram–based approach provides an important, new, and computationally convenient framework for investigating entanglement in biopolymers.

## Teaser

Grid diagrams grasp the principle of DNA topology simplification by DNA topoisomerases.

## INTRODUCTION

DNA topology is regulated by enzymes called topoisomerases. A class of these, known as type II topoisomerases (*1*), act on double-stranded DNA (dsDNA) molecules by introducing dsDNA breaks that are bridged by the bound enzyme (*2*). Subsequently, a distinct dsDNA segment (either from the same molecule or from another one) is passed through, and the cut is resealed (*1*). Since topoisomerases are of vital importance for the proper functioning of DNA replication (*3*) and of several other cellular processes (*4*), they are often used as targets for antibacterial and anticancer drugs (*5*, *6*). Although type II topoisomerase’s ability to cleave and reseal DNA molecules plays such a fundamental role, performing intersegmental passages on long polymers often results in the creation of nontrivial knots and catenanes (also known as links). DNA knots are damaging for the cell (*7*). Therefore, they should be quickly unknotted by DNA topoisomerases. Recent determination of human chromosome’s structure from single-cell HI-C data revealed that chromosomes can be knotted as well (*8*), as it was earlier proposed in (*9*). It has been observed in reactions performed in vitro that when type II topoisomerases act on randomly cyclized DNA molecules (i.e., molecules having the equilibrium level of knotting), the level of knotting decreases markedly (*10*). Thus, type II topoisomerases manifest a preference to unknot the DNA, and many biological explanations for this behavior have been proposed (*10*–*14*). Rybenkov *et al.* (*10*), in their paper establishing the concept of preferential unknotting by DNA topoisomerases, proposed that type II topoisomerases form clamps that actively slide along the DNA, concentrating DNA entanglements and thus facilitating DNA unknotting. Since the active sliding mechanism was not confirmed experimentally, the same group hypothesized later that topoisomerase creates a sharp bend in the DNA region that will be transiently cut during the reaction and that the creation of the bend directs the passage of the transported segment through the transient cut by passing from inside to outside of the bend formed by the DNA topoisomerase (*11*). More recently, it was hypothesized that type II topoisomerases are preferentially unknotting DNA because of their ability to specifically recognize and act on hooked juxtapositions of DNA segments (*14*).

Lattice-based simulations (*15*, *16*) and equilateral chain model simulations (*17*) have confirmed this hypothesis. However, these prior simulations, and previous investigations (*18*, *19*) of connectivity between neighboring knot spaces via single intersegmental passages, necessitated the use of computationally expensive randomization algorithms to ensure uniformity of sampling and to produce systems exhibiting global and detailed balance while undergoing a given type of intersegmental passages (as discussed in Results). Hence, our aim here is to propose a new, computationally convenient, purely topological, and intrinsically randomized framework to examine how the interconversion rates between different knot types depend on the local geometry of regions where the intersegmental passages occur.

Since the topology of all knots can be encoded by planar projections containing the information of over/underpassing segments (i.e., knot diagrams), we use planar diagrams and, more specifically, grid diagrams (*20*, *21*) for our analysis. We provide exact enumeration of the intersegmental passages between grid diagrams taken up to a certain complexity called the grid number (GN). This measure of complexity for a knot diagram can be easily related to the length of the polymer associated to the diagram; grids with grid number *n* can be thought of as polymers with circa 2*n* statistical segments (*22*). Our approach enables us to compute exact values for the distribution of knot types after an intersegmental passage between diagrams with GN less than 8. In the case of higher complexity diagrams, the complete enumeration is impractical, so we randomly sample the space of diagrams and determine the topological consequences of intersegmental passages. Random sampling is easy in this setting. This is because every grid diagram can be uniquely represented by a pair of *n*-tuples (i.e., by two strings of *n* natural numbers) (*21*), and there are several available algorithms to uniformly sample natural numbers [e.g., Python’s extensively tested random function (*23*)]. Thus, grid diagrams are ergodic (see the Supplementary Materials for more details).

Of course, to decrease the level of DNA knotting below the thermodynamic equilibrium, type II DNA topoisomerases have to perform some work that is possible thanks to adenosine triphosphatase activity of these enzymes. It has been proposed that adenosine triphosphate is used to give a unique direction of passage of the transferred DNA segment so that the transferred segment always passes from inside to outside of the bend formed by the segment cut and resealed during the reaction (*11*). In our simulations, all passages mimicking topoisomerase-mediated actions happening at hooked juxtaposition are performed as if the transferred subchain passes from inside to outside of the bend formed by the hooked juxtaposition.

We first apply the grid diagram approach to estimate the probabilities of passing from one knot type to another through the action of a hypothetical unbiased topoisomerase acting on circular DNA molecules of various lengths. We then test the consequences of passages occurring at “hooked juxtaposition” (*14*) and calculate the resulting knotting reduction factor.

## RESULTS

### Grid diagrams

**Fig. 1:**
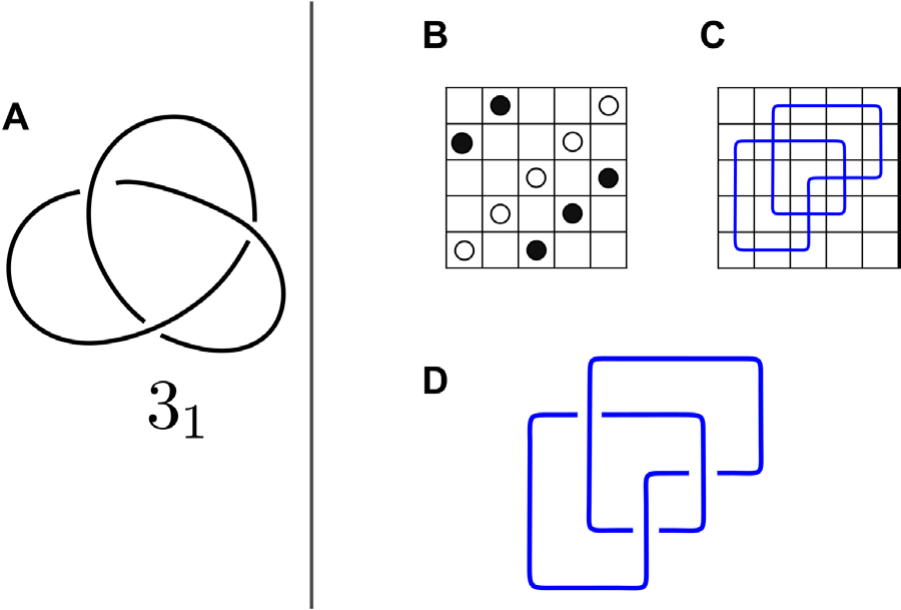
A standard knot diagram and a grid diagram of a knot. (**A**) Standard diagram of a left-handed trefoil knot. We use Alexander-Briggs’ notation of knots, where the first number indicates the minimal crossing number of a given knot type and the subscript number denotes its tabular position among all knots with that crossing number. Standard knot diagrams are scale free and therefore do not inform about the number of statistical segments of a knotted polymer that they represent. (**B** to **D**) Generation of a grid diagram of a trefoil knot. Grid diagram formalism requires that the square grid with *n* rows and *n* columns has exactly one segment in each row and column of the represented polygonal chain. A grid diagram of a knot configuration is generated in three steps. (B) Place 2*n* markings (dots) corresponding to ends of the modeled polygonal chain segments. Markings are placed following a “sudoku” rule, requiring that each column and each row of the grid contain exactly two markings in distinct squares. (C) Each pair of markings in the same row/column is connected by a segment. (D) For the segments that intersect, we follow the convention that the vertical segment passes over the horizontal segment.

Grid diagrams are a special kind of knot diagrams, first introduced in (*20*) and widely used in knot theory [see, e.g., (*21*)]. Grid diagrams consist of a square, planar, *n* × *n* grid (*n* is a natural number greater than 2), in which 2*n* markings are placed, corresponding to vertices of a piecewise linear curve representing a given knot. Each row/column of the grid must contain exactly two markings (see Fig. 1B), and two markings cannot occupy the same position. A knot diagram can be created from a grid diagram as follows: Connect by a segment any two markings on the same row or column, and impose every vertical strand to be overpassing. We remark here that the overpassing condition is not restrictive since any knotted configuration can be represented by a grid diagram (*21*). Just as for classical knot diagrams (*24*), there exists a finite set of moves that allows us to represent any deformation of the underlying curve (the knotted DNA molecule, in our case) in terms of local transformations of the grid [i.e., local displacement of the markings (*21*)]. Refer to the Supplementary Materials for more details on grid diagrams.

There is an immediate advantage when considering grid diagrams, as opposed to other ways of modeling knots (e.g., classical diagrams, lattice, or stick models): Each grid diagram can be efficiently described by a pair of permutations (that is, a pair of *n*-tuples) on *n* elements, determining the positions of the markings. The number *n* is a measure of complexity for grid diagrams, and it is called the grid number GN. A grid diagram of size *n* can be seen as corresponding to an equilateral polymer chain with 2*n* statistical segments [see, e.g., (*22*)]. For example, grid diagrams in GN 5 can be used to estimate the knotting probability of a polymer having the length of ∼10 statistical segments, which, in the case of DNA molecules, would correspond to ∼3-kb.

**Fig. 2:**
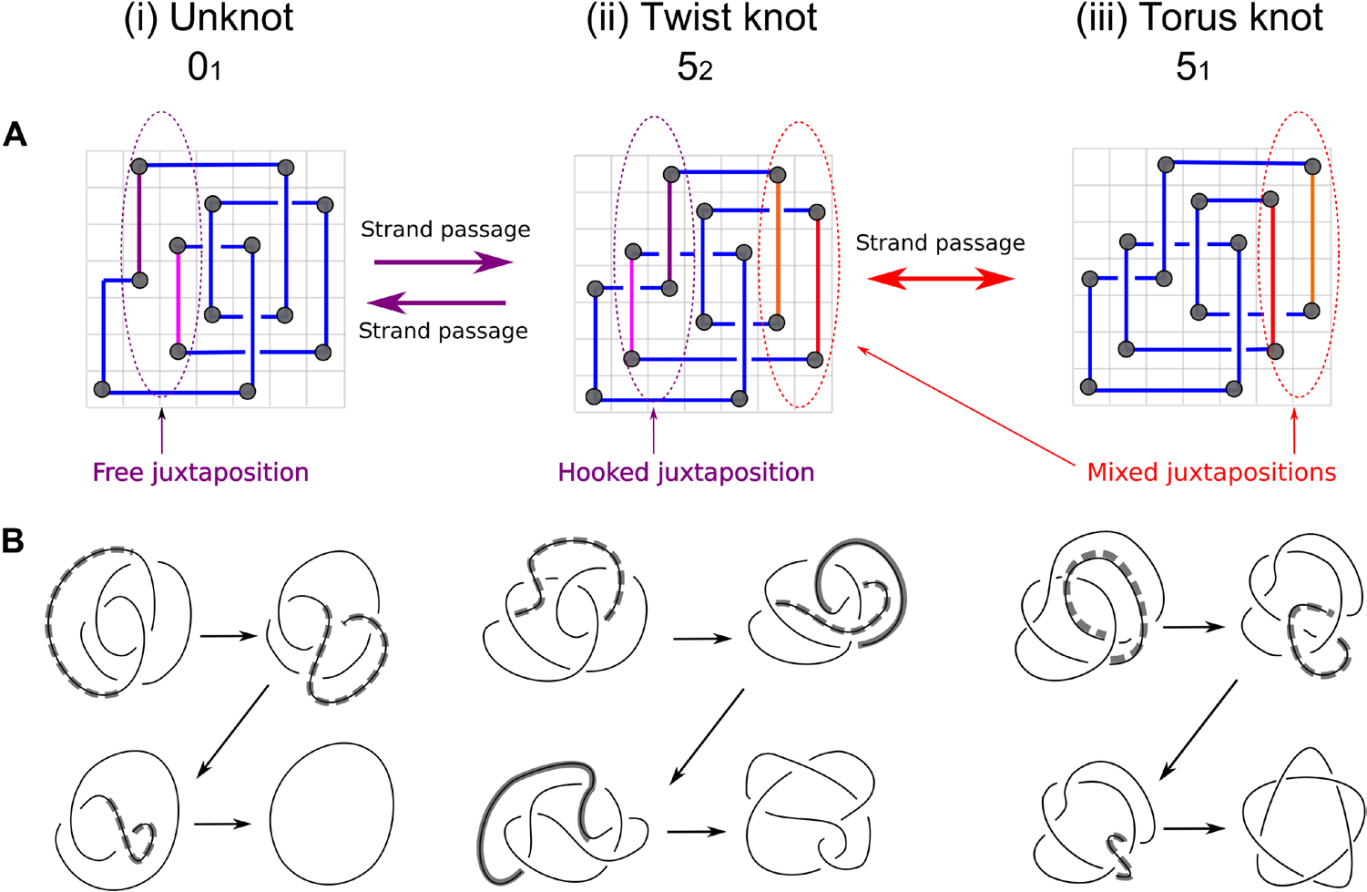
Interleaving commutations as a model for strand passages. (**A**) Intersegmental passages between grid diagrams representing the (i) unknot 0_1_, the (ii) twist knot 5_2_, and the (iii) torus knot 5_1_. Intersegmental passages are achieved by interleaving commutation of two consecutive rows/columns such that the interiors of their corresponding intervals intersect nontrivially but neither is contained in the other. The exchanged segments are highlighted in red and purple. When the area delimited by two parts of the diagram between two consecutive crossings forms a rectangle, a hooked juxtaposition is formed. A juxtaposition can be “strongly hooked” if the rectangle is a square. The diagram of 5_2_ contains one strongly hooked juxtaposition. Performing an interleaving commutation at that hooked juxtaposition transforms the 5_2_ into the trivial knot. This exchange transforms the hooked juxtaposition into a “free” one. Juxtapositions that are neither hooked nor free are called mixed. Performing an interleaving commutation at the highlighted mixed juxtaposition on 5_2_ transforms this knot into the 5_1_ (shown on the right). (**B**) The local deformations that transform the three grid diagrams in (A) into their respective standard diagrams. Thick gray lines highlight the arcs involved in the various deformations.

Local deformations and strand passages (i.e., intersegmental passages, thus, single actions of type II topoisomerases) are then realized by changing the pair of permutations. In particular, strand passages are achieved on grids by a process called interleaving commutation, which exchanges the positions of two adjacent and interleaved rows or columns, as described in Fig. 2A. This operation does not increase the complexity of the grid diagram, so it can be easily encoded. This combinatorial description of grid diagrams allows us to perform exact enumeration for a range of complexity, and theoretically, it allows computations with grids of arbitrary dimensions.

### Free and hooked juxtapositions

The part of a diagram between consecutive crossings can take different shapes, some of which correspond to the projection of a hooked juxtaposition. We call a hooked juxtaposition the part of a grid diagram in which the segments between two consecutive crossings geometrically form a rectangle [see Fig. 2 (ii)]. In analogy with the lattice models (*15*, *16*), we test the effects of geometrical selection of sites (where the intersegmental passage happens) on the topological outcome (see Fig. 2). By imposing that the crossing changes happen only at the specific local configurations resembling the hooked geometries (*17*), we can test the hooked juxtaposition hypothesis (*14*) that type II topoisomerases achieve disentanglement by performing strand passages only at hooked juxtapositions. We measure how much a juxtaposition is hooked using the area of such rectangle as a parameter: This quantification of hooked means that the larger the parameter value, the less the configuration is “hooked.” We call “strongly hooked” the juxtapositions in which the rectangle is the elementary square. Note that, as shown in Fig. 2, a strand passage occurring at a hooked juxtaposition transforms the geometric site into a “free” juxtaposition.

### Knot interconversion fluxes resulting from unbiased intersegmental passages are balanced for all realizable configurations in grids with GN 6

**Fig. 3:**
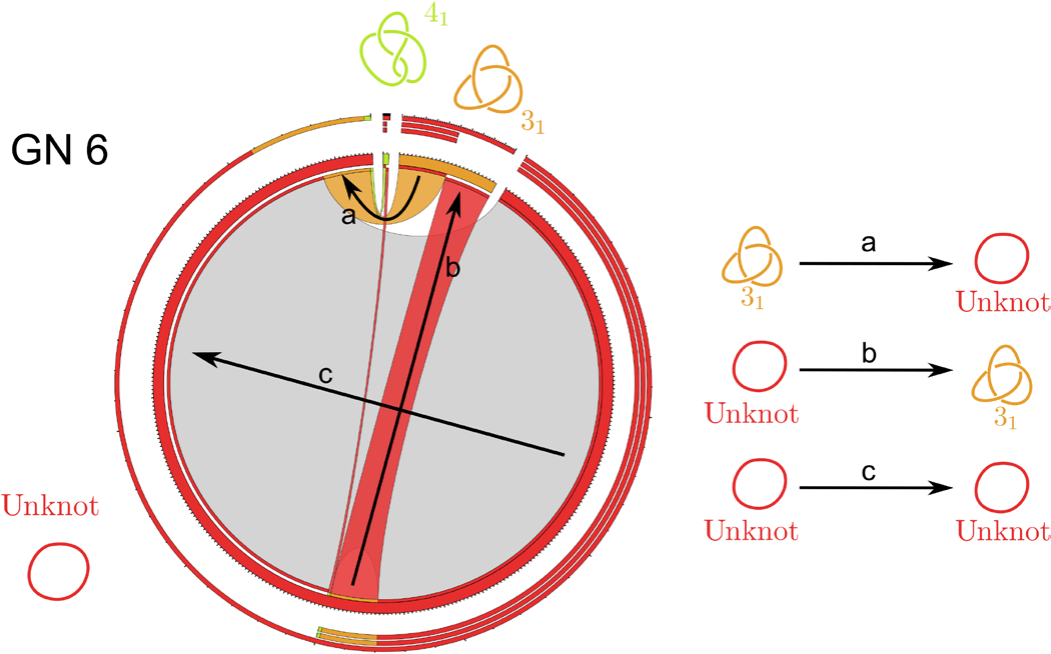
Visualization of strand passage–mediated knot interconversion fluxes using circos plots. The three layers of thin external arcs, progressing from inside to outside, represent outgoing, incoming, and total fluxes involving a given knot type, respectively. These external arcs are segmented to indicate how the respective fluxes were redistributed. The thickness of the (interior) chords connecting different knot types reflects the fraction of outgoing and incoming knot interconversion fluxes between given types of knots. The chords representing knot interconversion fluxes are colored as the knot type that these fluxes originate from, with the exception of chords starting and ending in the same knot type, which are in gray. These correspond to the fluxes resulting from strand passages not changing the knot type. The bases of the chords representing outgoing fluxes are colored according to the knot type that a given flux leads to. The bases of chords representing incoming fluxes are left white. The length of the various thick arcs around the circumference, colored as the corresponding knot diagrams, indicates the sum of fluxes outgoing from and incoming to a given knot type. The global observed flux in a given system is normalized to 1, which corresponds to the circumference of the circle.

The circos plot in Fig. 3 summarizes the data obtained in calculations where all configurations of knots (including trivial knots) that are realizable as grid diagrams with GN 6 undergo intersegmental passages resulting from unbiased interleaving commutations. The thickness of cords connecting arcs representing different knots is proportional to the interconversion fluxes connecting these knots. Each pair of knots *i* and *j* is connected by two cords that represent the interconversions of all the configurations representing the knot *i* into configurations representing the knot *j* and the interconversions of the knot *j* into the knot *i*. As can be seen, these incoming and outgoing cords connecting a given pair of knots have the same thickness at their starting and ending portions. Thus, for example, the cord representing the outgoing flux from trefoil knots to trivial knots (marked with an arrow “a”) has the same thickness as the cord representing outgoing flux from trivial knots to trefoil knots (marked with an arrow “b”). The circos plots that we used convey even more information about the knot interconversion fluxes, and the legend to Fig. 3 explains how this information is encoded.

### The configuration space of grid diagrams with GN ≤ 7

**Fig. 4:**
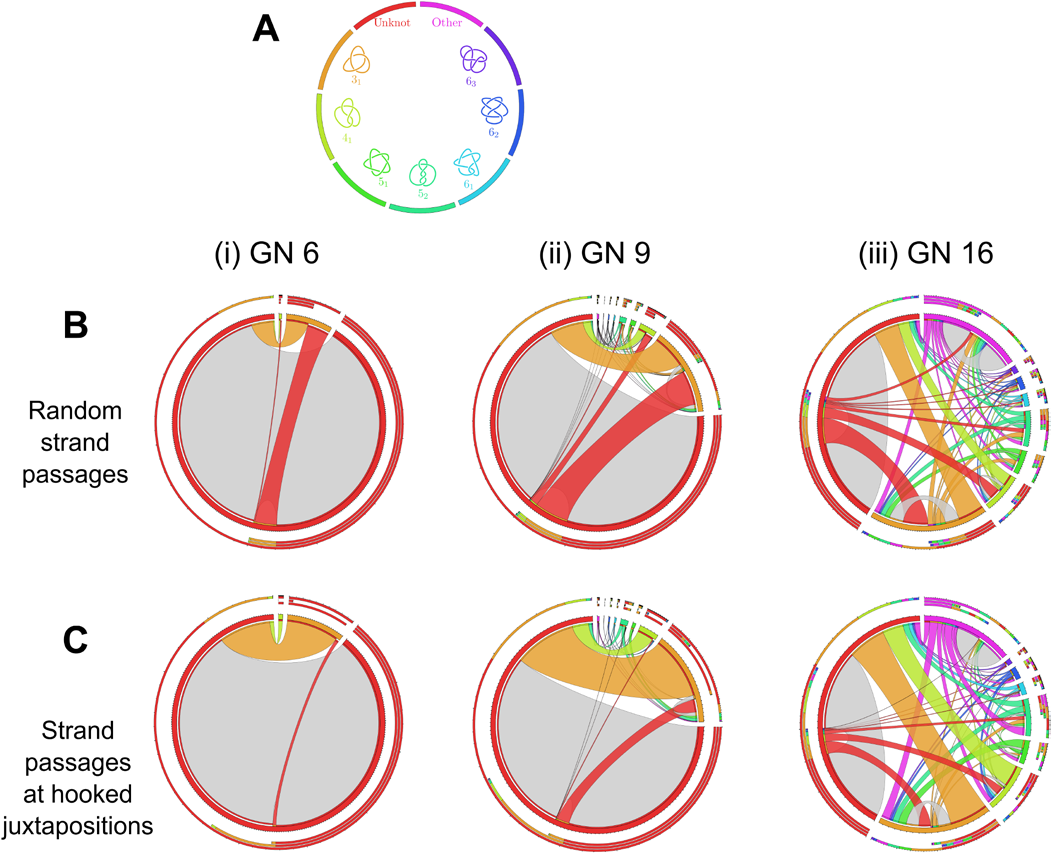
Knot interconversions occurring at hooked juxtapositions lead to preferential unknotting. (**A**) Color guide informing which colors correspond to which knots in circos plots shown in (B) and (C). A comparison of the topological consequences of unbiased strand passages (**B**) with the ones resulting from strand passages occurring only at strongly hooked juxtapositions (**C**). Comparison of circos plots for small grid diagrams (i) with those for larger grid diagrams (ii and iii) shows that as the system gets more complex, more types of knots are formed and they contribute stronger to the knot interconversion fluxes. In the circos plots representing fluxes resulting from unbiased strand passages (A), the incoming and outgoing fluxes connecting any pair of knots are of the same intensity. This indicates that the generated set of grid diagrams represents the topological equilibrium. When the same set of grid diagrams undergoes intersegmental passages involving only strongly hooked juxtaposition, the interconversion fluxes from the trefoil to the unknot are much more intense than the opposite fluxes. This effect is especially strong for smaller GNs.

There are a total of 1,859,118 different grid configurations with grid number GN ≤ 7, of which 1,773,114 are unknotted, 78,296 are (left- or right-handed) trefoils, 6014 are figure eight knots, 798 are 5_1_ torus knots, 882 are 5_2_ twist knots, and only 14 of them are 8_19_ knots. As an example, the unbiased adjacency table of knots with GN 6 is visualized in the leftmost circos plot on Fig. 4B (i). We observe that, in agreement with previous works (*18*, *19*), most (<91.7%) of the strand passages occurring in unknotted diagrams do not change the topology of these diagrams and, of those passages that change the topology, <94.5% transform the unknot into the trefoil.

The circos plots summarizing the sampling of GN 9 and 16 are shown in Fig. 4 (B and C, ii and iii). It is immediately apparent how the knot-type fauna becomes more variegated as the GN increases, with considerably higher occurrence of complex knots, and strand passage–mediated fluxes become more visible. In the exact enumeration of unbiased strand passages, the number of diagrams passing from the *i*-th knot type to the *j*-th knot type is equal to those passing from the *j*-th to the *i*-th. Thus, the fact that outgoing and incoming fluxes are equal shows that performing all the interleaving commutations does not introduce any selection bias. In higher GNs, we have to ensure that the sampling method enables us to describe the system at equilibrium. As mentioned in the Introduction and Materials and Methods, by performing every strand passage on each one of the configurations randomly sampled, we achieve detailed and global balance effortlessly. This can be seen from the circos plots of Fig. 4B (ii and iii), in which the sizes of the arcs representing incoming and outgoing fluxes of every pair of knot types correspond almost exactly.

### Evolution of the connectivity between knot spaces

**Fig. 5:**
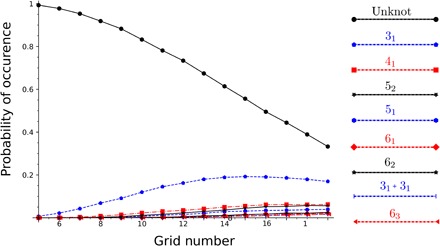
Occurrence probability of prime knots as a function of GN. The curves give the occurrence probabilities of knots with minimal crossing number up to 6, plotted as a function of the grid number. The occurrence probability of a given knot type is defined as the ratio between the configurations representing that knot type and the total number of configurations. We consider also the composite knots obtained as the connected sum of two trefoils.

**Fig. 6:**
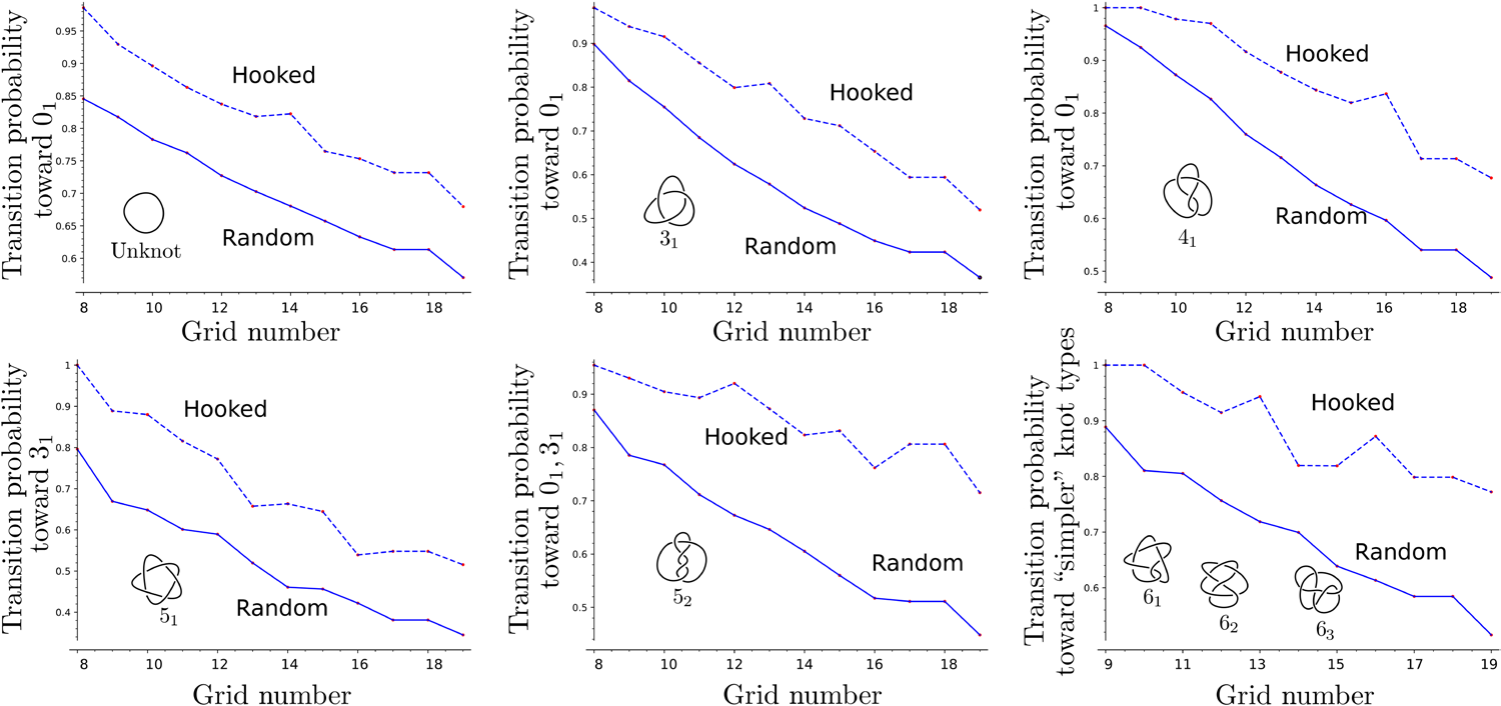
Topological simplification due to hooked juxtapositions. The plots show the transition probabilities of each knot type toward simpler knots, as a function of the grid number. In each plot, the dotted line refers to strand passages happening at strongly hooked juxtaposition, while for the other, we consider unbiased interleaving commutations. In the case of the unknot, the 3_1_ and the 4_1_, we consider only the unknotting probabilities. For the 5_1_ and the 5_2_, we consider passages toward the 3_1_ and toward the 3_1_ and the unknot, respectively. Last, for 6 crossings knots, we plot the transition probabilities toward knots with lower “length-over-diameter ratio” (thus, for the 6_1_, we consider only passages toward knots with crossing number less than or equal to 5, while for the 6_2_ and the 6_3_, we also consider passages toward the 6_1_ and toward the 6_1_ and 6_2_, respectively) (*37*).

It is well known (*25*, *26*) that for closed polymers, the probability that a configuration is unknotted decreases as the length of the polymer increases. The same behavior can be observed for grid diagrams. Figures 5 and 4 show how the configuration spaces of a knotted molecule evolve as the complexity given by the GN increases. The probability that a configuration of a given knot type is converted into another knot type is called the transition probability of the first knot type toward the second. Note that as GN increases, the probability of occurrence of unknotted conformations decreases monotonically, and therefore, the transition probabilities toward “simpler” knot types also decrease monotonically, as shown in Fig. 6. For example, the probability of passing from a trefoil diagram to an unknot passes from an initial value of almost 1 to about 0.4 as the grid size increases from GN 5 to GN 19. Unsurprisingly, for every GN, the transition probability for the unknot passing to a nontrivial knot is consistently the highest for the trefoil.

One may be tempted to sort knot types connected through a single-strand passage to another knot, in terms of the amount of observed strand passages toward the knot types in question, as discussed in (*18*) (there, they refer to this concept as “interface area” between knot spaces). In our setting, we can formalize the heuristic notion of knot closeness between two given knot types as the ratio between the occurrences of intersegmental passages leading to the interconversion between the two knot types and the total number of intersegmental passages that lead to interconversion between any two knot types.

As an example, the data discussed above suggest that the trefoil is the closest knot type to the trivial knot. The observation that these properties are maintained as the GN increases, coupled with the fact that our results are topological in nature (hence, independent of any geometric or physical feature of the model), indicates that these are intrinsic features of the various knot types. Figure 5 shows how the knot fractions change as the GN increases.

We observe that the trend is qualitatively similar to the case of equilibrated polygonal chains in three dimensions (3D) [see, e.g., figure 2 of (*27*)]. However, in polygonal chains formed by essentially 2D grid diagrams, knotting is stimulated as compared to 3D situation (*27*). In our case, the population of nontrivial knots starts to exceed 50% at GN 17, and every knot with minimal crossing number ≤ 13 can be represented by grids of size ≤12 (*21*). This highlights a further advantage of our framework. Namely, we are able to account for complex knot types while working with configurations of relatively small size. Further details on comparisons between our model and previous approaches on the topic are discussed in the Supplementary Materials.

### Topological simplification through geometric selection of sites and the knotting reduction factor

Figures 4 and 6 show how the knot interconversion fluxes resulting from intersegmental passages occurring once at every interleaved juxtaposition are affected by limiting intersegmental passages to interleaved juxtapositions that are strongly hooked. In the case of passages occurring once at every interleaved juxtaposition (Fig. 4B), the fluxes between any two knot types are equilibrated. This is best seen for small GNs, where we enumerate all possible configurations. In these cases, the number of observed interconversions from trivial knots into trefoil knots, for example, was exactly the same as the number of interconversions that changed trefoil knots into trivial knots. The probability that a given configuration of a trivial knot interconverted into a trivial knot was, for analyzed grid sizes, always substantially smaller than the probability that a given configuration of a trefoil knot converted into trivial knot. However, the number of realizable unknotted configurations was larger than the one of trefoil knots. The difference in the numbers of unknots and trefoils was such that the sum of all interconversions from unknots into trefoils was exactly the same as the sum of all interconversions from trefoils into unknots.

Circos plots presented in Fig. 4B correspond to a situation in which DNA circles with a given size are permitted to undergo random intersegmental passages until they reach an equilibrium. Such a system at equilibrium shows both global balance and detailed balance. (Global balance means that the fraction of DNA molecules forming any given knot type reaches its equilibrium level and will not show a tendency to increase or decrease that level, although one may observe some fluctuations. Detailed balance means that the interconversion fluxes between any two types of knots are the same in both directions when observed over a sufficiently long time.) Our modeled system, where all intersegmental passages realizable by interleaved commutations are allowed, shows a detailed balance of interconversion fluxes. Thus, it is evident that our system is at the equilibrium state.

Figure 4 shows what happens when the equilibrated set of configurations obtained after unbiased intersegmental passages is allowed to undergo further intersegmental passages but, this time, only occurring at strongly hooked juxtapositions. The generated interconversion fluxes are no longer balanced, and the fluxes from more complex knots toward simpler knots are stronger than the opposite fluxes. The ratio of these initial interconversion fluxes connecting trivial knots with trefoil knots describes how much the ratio of the number of trefoil knots and unknots would need to be diminished so that the system will reach its new equilibrium. The extent of this knotting diminution, also known as knot reduction factor, was measured experimentally for intersegmental passages mediated by the type II topoisomerase, topoisomerase IV (*10*), and for several simulated systems (*15*–*17*). In experimental studies by Rybenkov *et al.* (*10*) it was observed that upon many rounds of type II topoisomerase–mediated intersegmental passages, the amount of trefoil-forming DNA molecules was greatly reduced as compared to the amount that would be obtained after random intersegmental passages. The experimentally observed reduction of trefoil knot concentration was approximately 90-fold for 7-kb DNA circles and approximately 50-fold for 10-kb DNA circles (*10*). The knotted population seen in the experiments by Rybenkov *et al.* (*10*) for 10-kb DNA circles divides into approximately 3% trefoils and 97% unknots. A similar ratio in our model can be found in the range 5 ≤ GN ≤ 7. We therefore analyze all knot diagrams realizable in grids of those sizes to determine what is the knotting reduction factor resulting from passages occurring only at strongly hooked juxtapositions.

We began with the enumeration of passages occurring at all juxtapositions and observed that there were 6240 interconversions from configurations representing the trefoil knot to configurations representing the unknot and the same number of interconversions from unknots to trefoil knots. We then analyzed passages at hooked juxtapositions and observed that there were 1220 interconversions from configurations representing the trefoil knot to configurations representing the unknot and only 80 interconversions from unknots to trefoil knots. Therefore, to equilibrate the interconversion fluxes observed when passages are limited to strongly hooked juxtapositions, the proportion of trefoil knots to unknots would need to be diminished over 15 times as compared to situations where the regions of passages are not selected. This would happen after many rounds of passages limited only to hooked-intersegmental juxtapositions. Note that the 15-fold knotting reduction factor concluded from our simulations is strong, but it is still substantially smaller than 50- to 90-fold knotting reduction factor observed experimentally (*10*).

Earlier simulation studies by Burnier *et al.* (*17*) showed that the more a juxtaposition is hooked, the larger the corresponding knotting reduction factor. In that study, a 23-fold knotting reduction was observed for hooked juxtapositions with acute angles smaller than 25°, whereas in our model, we are limited to right angles between segments forming hooked parts of the polymer. However, in analogy to what was discussed in (*17*), we investigated how the knotting reduction factor changes as the size of the rectangle area enclosed by the interleaving strands in hooked juxtapositions is decreased. For each GN, the knot reduction factor increases as this area decreases, reaching its maximum when the area enclosed by the interleaving strand in hooked juxtapositions reaches its minimum, which is equivalent to an elementary square of the lattice (see fig. S4). It is possible that the effective bending in hooked juxtapositions interacting with the type II topoisomerase, topoisomerase IV, is stronger than the one that we can model using grid diagrams. Our model can nevertheless capture the principle of knotting reduction resulting from geometrical selection of sites for intersegmental passages.

In experiments performed by Rybenkov *et al.* (*10*), 7-kb-long DNA circles showed a knotting reduction factor nearly two times higher than 10-kb-long DNA circles. The inverse relation between the knotting reduction factor and the size of circular DNA molecules stems naturally from the mechanism proposed in (*14*), in which a higher ratio between the bending rigidity and the size of DNA molecules corresponds to a higher type II topoisomerase–mediated knot reduction factor. This effect was confirmed in simulation studies using polygonal knots in the 3D lattice model (*15*, *16*).

As our model is essentially a 2D model, it is of interest to analyze how knot reduction factor changes with the grid size. For grid diagrams with GN 5 (where there are 10 diagrams forming the trefoil knot), the knotting reduction factor connected to intersegmental passages at hooked juxtapositions was infinite. All 10 diagrams of the trefoil knots had hooked juxtapositions, and all passages occurring at these juxtapositions converted the trefoil knot to the unknot. On the other hand, all passages occurring at strongly hooked juxtapositions in unknotted diagrams did not result in a change of topology. As already mentioned, for GN 6, the knotting reduction factor was approximately 15. Analysis of diagrams in GN 7 showed that the selection of sites results in a knotting reduction factor of about 8. Therefore, we can conclude that planar grid diagrams adequately capture the experimental observation that knotting reduction by type II topoisomerases is strongest when acting on short DNA molecules (*10*).

## DISCUSSION

Grid diagrams provide a new tool to investigate knot adjacency and the configuration space of knots. They provide a way to examine the statistical and probabilistic properties of knotted polymers without the need to rely on initial configurations as starting points. Thanks to their intrinsic combinatorial definition, grid diagrams allow us to uniformly sample the space of simple and even complex knots in arbitrary large grid diagrams. This is in contrast with analysis in the equilateral chain model and lattice model, for which uniformity of sampling for long polymers is computationally expensive.

Although the mathematical formalism of grid diagrams limits the number of possible configurations of modeled chains, as compared to off-lattice situations, one can use grid diagrams to study the effects of various geometrical constraints on the consequences of modeled topoisomerase-mediated passages. We showed that passages occurring within hooked juxtapositions with the smallest area enclosed by the interleaving strands were most effective in topology simplification. This was analogous to the situation observed in the lattice modeling (*17*). The effects of twisted hooked juxtapositions on simplification of knots [(*28*); see also (*29*)] can be addressed by observing the consequences of passages within a tight “diagonal row” of juxtapositions resembling a plectoneme in supercoiled DNA. To study the effects of the juxtaposition angle considered earlier [(*30*, *31*); see also (*32*)], one may observe the consequences of passages in most tight juxtapositions, which are not immediately followed by next tight juxtapositions, as such juxtapositions in a 3D situation naturally predispose the contacting segments to be perpendicular to each other. On the other hand, the hooked juxtapositions with a large area of the rectangle enclosed by the interleaving strands would correspond in a 3D situation to junctions with a larger angular freedom of the juxtaposition.

Furthermore, since complex knot types (i.e., with high minimal crossing number) arise in relatively low grid number with significant percentages, this allows us to easily investigate statistical features of knotted polymers in a varied population of knots.

For these reasons, we believe that the grid diagram approach provides a complementary and valid reference system that helps to better understand mechanisms of action of various DNA topoisomerases, strand passage events, and even polymers in general.

## MATERIALS AND METHODS

### Modeling of dsDNA molecules and of type II topoisomerase–mediated actions using knot diagrams

Although DNA forms a double helix, for such topological aspects as DNA knots, DNA helicity can be frequently neglected and the formed knot can be recognized from a projection of the axial path of analyzed DNA molecules (*1*). These projections can be conveniently represented as grid diagrams (see Figs. 1 and 2) (*21*). Grid diagrams encode the information of which arc is overpassing and which is underpassing at each crossing (see Fig. 1A). A single action of a type II topoisomerase corresponds to performing a crossing change in the diagram (that is, exchanging the over- and underpassing arcs), as described in (*33*). It is well known that any knot type admits infinitely many different diagrams (*24*), so in this setting, we can model the configuration space of a circular DNA molecule undergoing the action of a type II topoisomerase as a directed network. In this network, the vertices are the 2D projections of the knotted configurations (i.e., the diagrams), and these vertices are connected through directed edges, each representing a single crossing change (i.e., an action of type II topoisomerase). A subspace of the network is formed by those vertices that correspond to diagrams representing a same knot type, and two knot types whose corresponding subspaces are connected by directed edges are called adjacent. More details on this network are available in the Supplementary Materials.

### The configuration space as a network

In our investigation, we considered grid diagrams with complexity between 5 (the minimal GN in which nontrivial knots appear) and 20. We created the network of these grid diagrams where two grids of the same GN are connected by a directed edge if it is possible to transform the first diagram into the second via a single strand passage (i.e., an interleaving commutation). For each GN, the network of grid diagrams has finitely many vertices and edges. The strand passage–mediated flux (or knot interconversion flux) from a knot type to another is the union of directed edges in the network going from the subspace corresponding to the first knot type into the subspace of the second one. The intensity of the flux is proportional to the number of these directed interconversion edges. We performed our analysis by enumerating knot conformations and by counting unbiased and hooked strand passages between conformations of given knot types. Imposing that the strand passages happen only at hooked juxtapositions changes the shape of the network of configurations. The subspaces corresponding to simple knot types become more preferable than the others, as we discussed in Results. Since the GN correlates with the length of the underlying closed polymer, the configuration space changes as GN increases in value (see Fig. 5), which is similar to models that take length as a parameter (*27*). (We remark that our model, being purely topological, does not consider such physical quantities such as temperature. In other words, the model is temperature independent, and each conformation is assigned the same statistical weight.)

### Statistical analysis: Exact grid enumeration

We began our analysis by enumerating all grid diagrams with grid number 5 ≤ GN ≤ 7. This was achieved using a Sage (*34*) program by listing all possible pairs of permutations of length 5 ≤ *n* ≤ 7 and keeping the ones representing knot diagrams. The topological state (i.e., the underlying knot type) of every configuration was determined using a combination of knot invariants [knot polynomials, determinant, and signature (*24*)]. We then performed every possible crossing change from each configuration. We summarized the data in a square table, the unbiased adjacency table, in which the (*i,j*)-th entry represents the number of strand passages from the *i*-th knot type to the *j*-th knot type. The adjacency table was then visualized using circos plots (see Fig. 3) (*35*). The previous process was then repeated, this time, by allowing only strand passages to occur at hooked juxtapositions (see Fig. 2). While the unbiased adjacency table is symmetric, the hooked one presents a strong unknotting preference, as discussed in Results.

### Statistical analysis: Sampling

Listing all possible configurations before and after strand passages provides information about adjacency between different knot types. The number of configurations grows superfactorially with GN [there are *n*/2((*n* − 1)!)^2^ different configurations of grid number *n*; see (*36*)], and the number of admissible crossing changes increases more than linearly with the complexity. Thus, an exhaustive computation for higher values of GN becomes quickly infeasible, so the investigation on diagrams with higher grid number was performed through random sampling. Since a grid diagram is completely determined by a pair of permutations defining the positions of the markings, uniformity of sampling was automatically built in our model. We only relied on the effectiveness of Python’s extensively tested random() function (*23*). We considered all knots whose minimal crossing number is less than or equal to 8 (labeling the more complex ones collectively as “other”) since these are the most commonly identified DNA topoisomers. We randomly sampled configurations of GN 8 and then performed every admissible strand passage (achieved by interleaving commutations; see Fig. 2A). After computing the starting and resulting knot types, we obtained an unbiased adjacency table, as before. We then compared the unbiased sampled table with the hooked sampled table obtained by restricting to strand passages happening at hooked juxtapositions. The same process was repeated for every grid number, for 8 ≤ GN < 20.

## Supplementary Material

http://advances.sciencemag.org/cgi/content/full/6/9/eaay1458/DC1

Download PDF

Grid diagrams as tools to investigate knot spaces and topoisomerase-mediated simplification of DNA topology

## SUPPLEMENTARY MATERIALS

Supplementary material for this article is available at http://advances.sciencemag.org/cgi/content/full/6/9/eaay1458/DC1 Supplementary Materials and Methods Computations and results Fig. S1. Local isotopy and crossing change. Fig. S2. The network of knot diagrams. Fig. S3. Grid diagrams. Fig. S4. Side-by-side comparison of circos plots in the 2D and 3D models. Fig. S5. The knot reduction factor increases with the tightness of hooked juxtapositions. References (*38*, *39*)

## Appendix

View/request a protocol for this paper from *Bio-protocol*.
