# Supplementary material for: Grid diagrams as tools to investigate knot spaces and topoisomerase-mediated simplification of DNA topology
Source: Sci Adv. 2020 Feb 26;6(9):eaay1458. doi: 10.1126/sciadv.aay1458 (PMC7043919; doi:10.1126/sciadv.aay1458)
Supplement: http://advances.sciencemag.org/cgi/content/full/6/9/eaay1458/DC1 [file supp_6_9_eaay1458__index.html]

Science Advances | Science AdvancesAAASSearchScience AdvancesMenu

## Supplementary Materials

**This PDF file includes:**

- Supplementary Materials and Methods
- Computations and results
- Fig. S1. Local isotopy and crossing change.
- Fig. S2. The network of knot diagrams.
- Fig. S3. Grid diagrams.
- Fig. S4. Side-by-side comparison of circos plots in the 2D and 3D models.
- Fig. S5. The knot reduction factor increases with the tightness of hooked juxtapositions.
- References (*38*, *39*)

Download PDF

**Files in this Data Supplement:**

- Adobe PDF - aay1458\_SM.pdf
