# Supplementary material for: Grid diagrams as tools to investigate knot spaces and topoisomerase-mediated simplification of DNA topology
Source: Sci Adv. 2020 Feb 26;6(9):eaay1458. doi: 10.1126/sciadv.aay1458 (PMC7043919; doi:10.1126/sciadv.aay1458)
Supplement: Download PDF [file aay1458_SM.pdf]

Science Advances
AAAS

advances.sciencemag.org/cgi/content/full/6/9/eaay1458/DC1

Supplementary Materials for

Grid diagrams as tools to investigate knot spaces and topoisomerase-mediated simplification of DNA topology

Agnese Barbensi, Daniele Celoria, Heather A. Harrington*, Andrzej Stasiak*, Dorothy Buck*

*Corresponding author. Email: harrington@maths.ox.ac.uk (H.A.H.); d.buck@bath.ac.uk (D.B.); andrzej.stasiak@unil.ch (A.S.)

Published 26 February 2020, *Sci. Adv.* **6**, eaay1458 (2020)
DOI: 10.1126/sciadv.aay1458

This PDF file includes:

Supplementary Materials and Methods
Computations and results
Fig. S1. Local isotopy and crossing change.
Fig. S2. The network of knot diagrams.
Fig. S3. Grid diagrams.
Fig. S4. Side-by-side comparison of circos plots in the 2D and 3D models.
Fig. S5. The knot reduction factor increases with the tightness of hooked juxtapositions.
References (*38*, *39*)

Materials and Methods
THE NETWORK OF CONFIGURATIONS

Knots and their diagrams. A *knot* is a smooth embedding of the unit circle S^1 into the 3-dimensional Euclidean space. We will call knots also the images of such embeddings. Thus, a knot is a closed curve in space. Two knots and are *equivalent* if their corresponding embeddings are related by an *ambient isotopy* (for a definition see (24)). This means that two knots K_1 and K_2 are equivalent if and only if the first curve can be smoothly (*i.e.* without performing cuts) deformed into the second one. A knot is called *trivial* (we will refer to it as the *unknot*) if it is equivalent to the standard embedding (*i.e.* the unknotted one) of the unit circle S^1 into the 3-dimensional Euclidean space.

We will often call *knot types* the equivalence classes of knots under the ambient isotopy equivalence relation. Analogously, we call a *link* an embedding of a disjoint union of *n* circles into the sphere. The image of each circle is called a *component* of the link, and a *link type* is the equivalence class of such an embedding, up to ambient isotopy. Thus, a knot is a link with only one component.

Knots are often studied through their *diagrams*. A diagram for a knot *K* is a projection of the knot into a 2-dimensional plane or sphere, such that the only singularities are transverse double points. Each intersection point is called a *crossing*, and it is endowed with the information of which strand is passing over (for more details and precise definitions see (24)). Every knot type admits infinitely many diagrams representing it. Moreover, every knot diagram has a finite number of crossings (21, Ch. 2).

Different knot types are distinguished using *knot invariants*. A knot invariant is a map that associates an "algebraic" object (*e.g.* a number or a polynomial) to a knot, taking the same value on equivalent knots. Many simple knot invariants can be defined using diagrams. For example, the *crossing number* of a knot *K* is defined as the smallest number of crossings of any diagram of the knot.

A *planar isotopy* can modify locally a knot diagram by slightly moving an arc as in fig. S1 (**A**), or by displacing a whole diagram, without creating or removing any crossing. We consider two diagrams to be *equivalent* if they differ only by a planar isotopy. We will often refer to a specific diagram as a *configuration* of the corresponding knot. Strand passages between different configurations are modelled as crossing changes on the respective diagrams (see fig. S1 (**B**)). Note that performing a crossing change might result in changing the knot type represented by the diagram.

The network of knot diagrams. We can represent the set of configurations of knots as a network (*cf.* (38)) in which:

- the vertices are knot diagrams (up to planar isotopy);
- the edges represent crossing changes (*i.e.* two vertices are connected by an edge if and only if they differ by a single crossing change).

There are infinitely many different knot types and for each knot type there are infinitely many different diagrams. Thus, this network is infinite. However, since each diagram has finitely many crossings, each vertex has only finitely many edges emanating from it. We show as an illustration a small part of this network in fig S2.

The network is partitioned in knot-subspaces. Each subspace is formed by those vertices corresponding to diagrams representing the same knot type. In fig. S2, a portion of the subspaces for the trefoil knot and the trivial knot are shown. Given two subspaces, we call the *flux* between them the union of all the edges connecting a vertex of one subspace with a vertex of the other.

Grid diagrams. Grid diagrams are a special kind of knot diagrams that provide an easy and combinatorial way to represent knots and links, first introduced in (20) (see also *e.g.* (21, Ch. 3), for definitions and results).

Definition 1 *A grid diagram is a n×n matrix G, together with two sets of n markings, denoted by* $\boldsymbol{X} = \{X_{0,\ldots,}X_{n-1}\}$ *and* $\boldsymbol{O} = \{O_{0,\ldots,}O_{n-1}\}$*. Each row and and each column of the matrix contains exactly one X and one O marking. A link diagram can be retrieved from a grid by connecting the X with the O in each row and column. In the crossings we let the vertical strand always pass over the horizontal one. The size of the matrix is a natural number n≥2, called the* grid number *GN of G.*

An example of a grid diagram representing the trefoil knot is shown in fig. S3 (**A**). A grid diagram can be described by two permutations $\sigma_{\boldsymbol{X}}$ and $\sigma_{\boldsymbol{O}}$. If there is a marking in the intersection of the *i*-th column and the *j*-th row, then $\sigma_{\boldsymbol{O}}$ maps *i* to *j*. The number of disjoint cycles in which the permutation $\sigma_{\boldsymbol{X}} \cdot \sigma_{\boldsymbol{O}}^{-1}$ splits is the number of components of the corresponding link diagram (link diagrams are defined exactly as knot diagrams) (21). In particular, if the number of cycles is 1, the diagram represents a knot.

Theorem 1.2 (*21, Ch. 3) Any link L can be represented by a grid diagram.*

In this setting, strand passages (*i.e.* crossing changes) are modelled as local moves on the grid. These moves are called *interleaving commutations*, see fig. S1 (**B**).

Definition 3 *Fix two consecutive columns (or rows) in a grid diagram G, such that their corresponding intervals intersect nontrivially, but neither is contained in the other. Let G' be the grid obtained by swapping these two columns (or rows). We say that the grid diagrams G and G' are related by an* interleaving commutation*.*

Theorem 1.4 (21*) If two grid diagrams G and G' are related by an interleaving commutation, their corresponding knot types K and K' are related by a crossing change.*

Grid diagrams are ergodic. Let G_N denote the set of grid diagrams of dimension N, for some N>>0, and likewise, denote by K_N the set of knot types represented by elements in G_N. Each element in G_N is uniquely determined by a pair of N-permutations, which we assume can be randomly sampled using python's *random()* function (23). In this context, ergodicity of our sampling population means that a given knot type $K \in K_N$ will be sampled with probability $\#\{G \in G_N \mid G \text{ represents } K\}/|G_N|$. This is equivalent to asking that the set of pairs of permutations P giving us grids in the set $S = \{G \in G_N \mid G \text{ represents } K\}$ is sampled with probability #{P yielding a grid in S}/(total number of pairs of permutations). This is indeed the case if we are considering the uniform probability on the set of pairs of permutations, which is equivalent to requiring that the *random()* function is indeed random, and hence ergodic.

The networks of grid diagrams. We can repeat the ideas from the previous section using grid diagrams instead of regular diagrams. In this case, we have an infinite network whose vertices are grid diagrams, and edges represent interleaving commutations. Again, since the number of interleaving row/columns of a grid diagram is bounded by a function of the grid number, every vertex has finitely many edges emanating from it.

Note that interleaving commutations do not change the size (the grid number *GN*) of a diagram. Thus, we can split the network of grid diagrams into a collection of finite components having grids with the same size as vertices.

More precisely, call G the network of grid diagrams. Then G, can be written as the disjoint union of G_n , for every n >1, where each G_n is the network of grid diagrams with *GN n*, connected by edges representing interleaving commutations (Note that we start from $n = 2$ since grid diagrams are not defined for $n = 1$. However, $n = 5$ is the minimum grid number that allows grid diagrams representing nontrivial knots. This is why in our computations we start from $n = 5$ instead.).

Since for every *n* there are finitely many possible grid diagrams having *GN n*, each network is finite. In particular, in each the flux between any two subspaces is made up by finitely many edges.

The specific nature of grid diagrams allows us to differentiate between different local geometries, as explained in the Materials and Methods of the main manuscript. Imposing that strand passages (*i.e.* interleaving commutations) happen only at specific local geometries (such as hooked juxtapositions, see Material and Methods in the main manuscript) induces an orientation of the edges, hence the network is directed.

Computations and results

A pair of permutations on *n*-elements σ_X and σ_O, represent a grid diagram for a link whenever $\sigma_X(i) \neq \sigma_O(i)$ for all $i=0,\ldots,n-1$. Moreover, by checking the number of cycles in $\sigma_X \cdot \sigma_O^{-1}$, we can tell whether it represents a grid diagram for a knot.

Exact enumeration. We generate all possible pairs of permutations on *n* elements for *n*=5,6,7, and check whether they represent a grid diagram of a knot.

For each grid, we determine the corresponding knot type using a combination of several knot invariants (Alexander and Jones polynomials, determinant, and signature, see (24) for definitions). We only consider knot types whose crossing number is ≤ 8, and we label the configurations representing more complex knot types as *other*. We then perform all possible interleaving commutations from the grid, and compute the knot type for each of the resulting grid diagrams.

We collect the data in a square matrix, called the *(unbiased) adjacency table*.
The (*i*,*j*)-entry of this table is the number of computed interleaving commutations from grids representing the knot type *i* to grids representing the knot type *j* (the knot types are ordered following the Alexander-Briggs' notation (24)). Note that since we are performing an exact enumeration, this table is symmetric.

We then restrict to interleaving commutations that occur only at hooked juxtapositions (see main manuscript, Materials and Methods). Recall from the main manuscript that we measure how much a juxtaposition is hooked using the area of such a rectangle as a parameter: the larger the parameter value, the less the configuration is hooked.

Again, we collect the information in several adjacency tables for passages at hooked juxtapositions. More precisely, for each grid number *GN* we created $GN-3$ (hooked) adjacency tables. Each of these tables accounts for passages happening at juxtapositions where the area of the rectangle is $\leq l$, where $1 \leq l \leq GN-3$. The probability that a configuration representing a specific knot type is transformed into an unknotted one by an interleaving commutation increases as we consider hooked juxtapositions with shorter edges.

The complete code is available at (39).

Sampling. For each *GN* within the range 8-19 (refer to the main manuscript for the chosen range of grid numbers) we proceed by randomly sampling a pair of permutations, and checking if it represents a valid knot grid diagram. We collect a certain number of configurations, which depends on *GN* (for higher *GN* we performed 16 iterations of the process, each time sampling 1000 configuration; for smaller *GN* we iterated the process twice, sampling 8000 configurations each time). We then proceed as done for the "Exact enumeration" on this sample. Again we produce several adjacency tables (both unbiased and hooked). The complete code is available at (39, Sampling.sage).

Data. The adjacency tables are available at (39). Each file contains the data relative to a specific grid number *GN* (or range of *GN*s), for a total of 16000 configurations sampled in each *GN*. In each file, for each iteration, we write:

- the *GN*;
- the total number of configurations considered (*e.g.* the number of sampled configurations plus the number of configurations reached through strand passages);
- the number of sampled configurations;
- the distribution of knot types in the sample;
- the distribution of knot types with respect to the total number of configurations considered;
- a list of adjacency tables, starting from the unbiased one, followed by the hooked ones. The hooked adjacency tables are ordered from less to more hooked.

Comparisons with previous models. Previous investigations of the connectivity between neighbouring knot spaces via single intersegmental passages have been often performed in the equilateral chain model (see *e.g.* (18,19)). As mentioned in the Introduction of the main manuscript, grids with grid number *n* can be thought of polymers with circa 2*n* statistical segments (22). As an example, we compare our data with the one obtained in (18) on polygonal chains of 33 segments. Figure S4 shows side-by-side the circos plot for 3D models of equilateral chains composed of 33 segments **(B)** and for grid diagrams with *GN* 12 (thus, that can be thought as a polymer with 24 segments) **(A).** It is apparent that the two circos plots are similar but not identical. Indeed, the transition probabilities between different knot types computed in the two models are very close. For example, the unknotting probabilities of all the nontrivial knot types

considered are almost identical in the two plots. However, as explained in the main manuscript, in 2D grid diagrams we observe a much higher probability of knotting that in 3D walks, for the same number of segments. Note how in both systems the passages from a knot A to knot B are observed with the same probability as passages from knot B to knot A. This detailed and global balance condition shows that both models are suitable to study equilibrium probability of knotting in a given system.

As mentioned in the main manuscript, we investigated how the knotting reduction factor changes as the size of the rectangle area enclosed by the interleaving strands in hooked juxtapositions is decreased. This was done by considering each time passages happening at juxtapositions where the area of the rectangle is $\leq l$, where l varied between $GN-3$ and 1.
In analogy with what discussed in (17) with the angle of a juxtaposition, in every *GN*, the knot reduction factor increased as the area of the rectangle decreased. As an example, fig. S5 shows the case of *GN* 12.

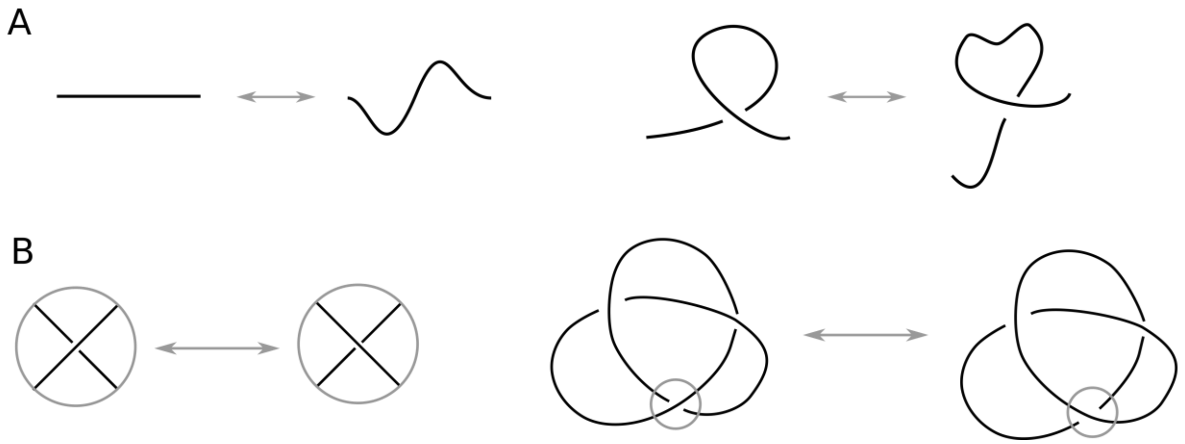

Fig. S1. Local isotopy and crossing change. (**A**) Two examples of a local planar isotopy acting on an arc. (**B**) Changing a crossing in a knot diagram. On the right, two diagrams differing by a crossing change

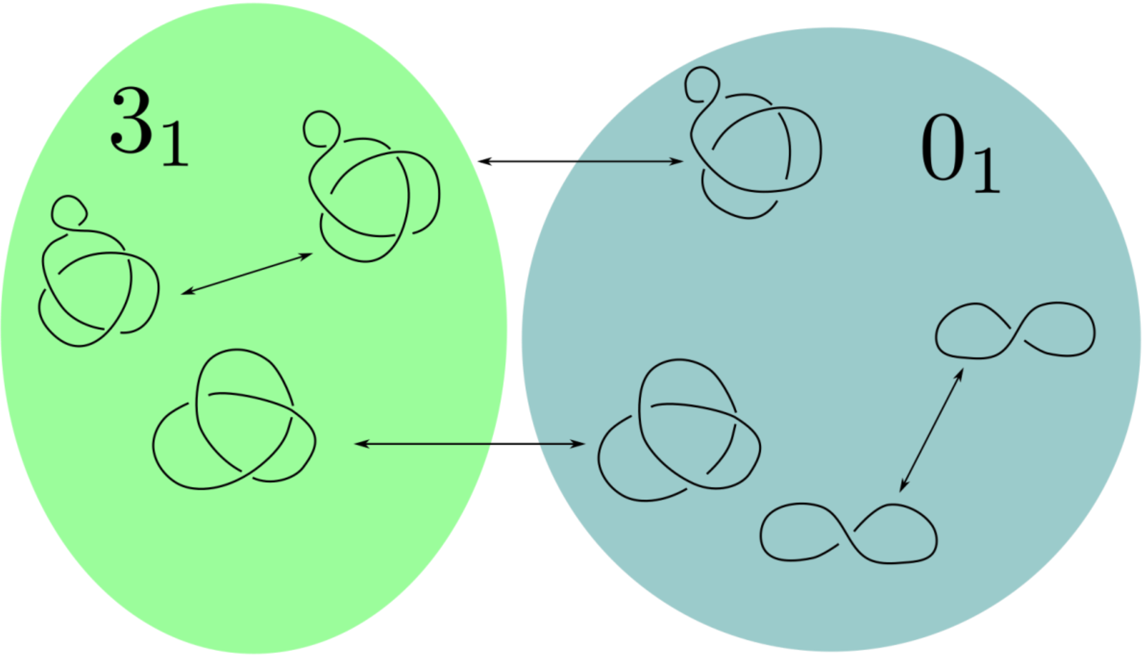

Fig. S2. The network of knot diagrams. A part of the network of configurations, consisting of 7 vertices and 4 edges. A part of the trefoil subspace (on the left) and of the trivial knot subspace (on the right) are shown. Edges represent crossing changes. Edges connecting vertices in the trefoil subspace with vertices in the trivial knot subspace form the flux between the trefoil and the unknot.

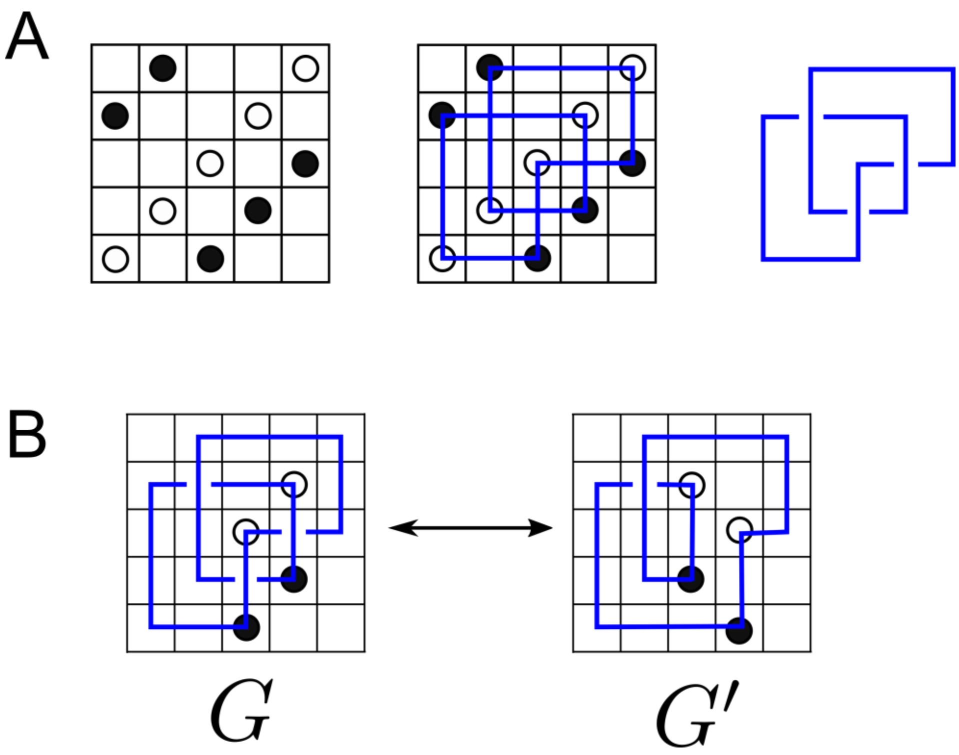

Fig. S3. Grid diagrams. (**A**) A grid diagram representing the trefoil knot. Here the black circles represent the *O*-markings and the white circles the *X*s. It follows that $\sigma_{\mathbf{X}} = (0,1,2,3,4)$ and $\sigma_{\mathbf{O}} = (3,4,0,1,2)$. (**B**) An interleaving commutation between the grid diagrams *G* and *G*'.

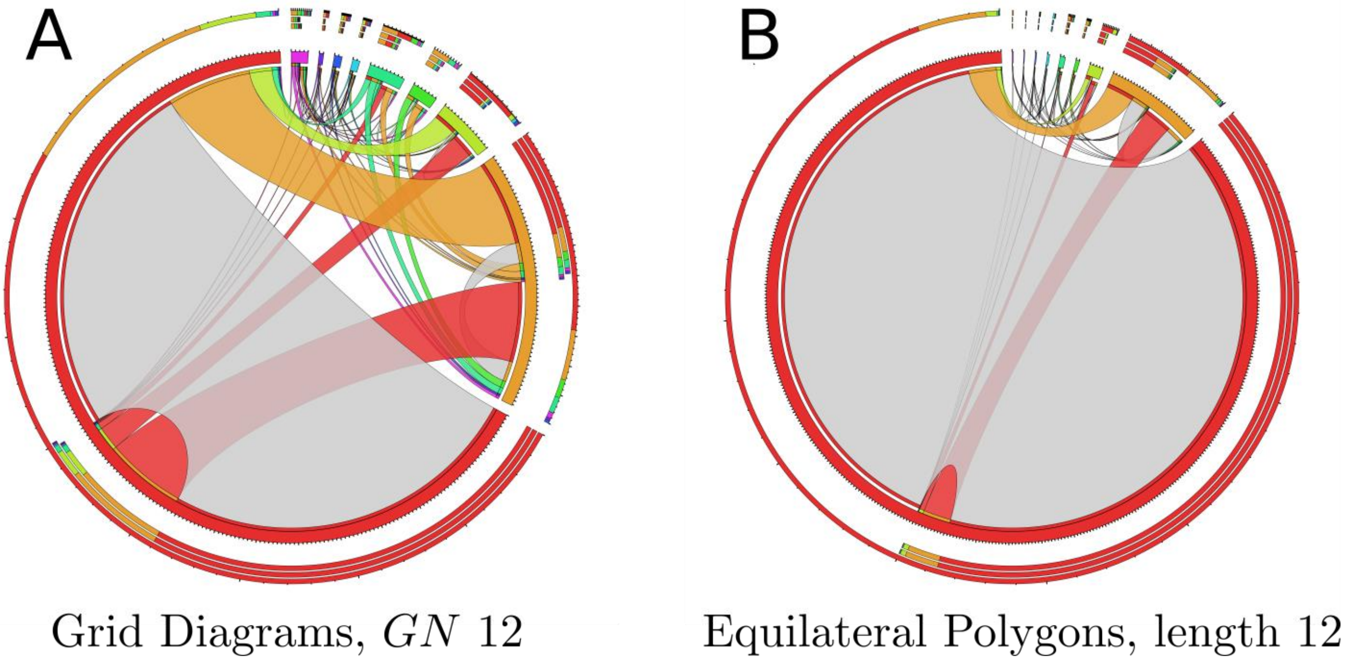

Fig. S4. Side-by-side comparison of circos plots in the 2D and 3D models. (**A**) Circos plots representing fluxes resulting from unbiased strand passages in *GN* 12. (**B**) Circos plot summarising the data of (18), Table 1, of equilateral polygons composed of 33 segments.

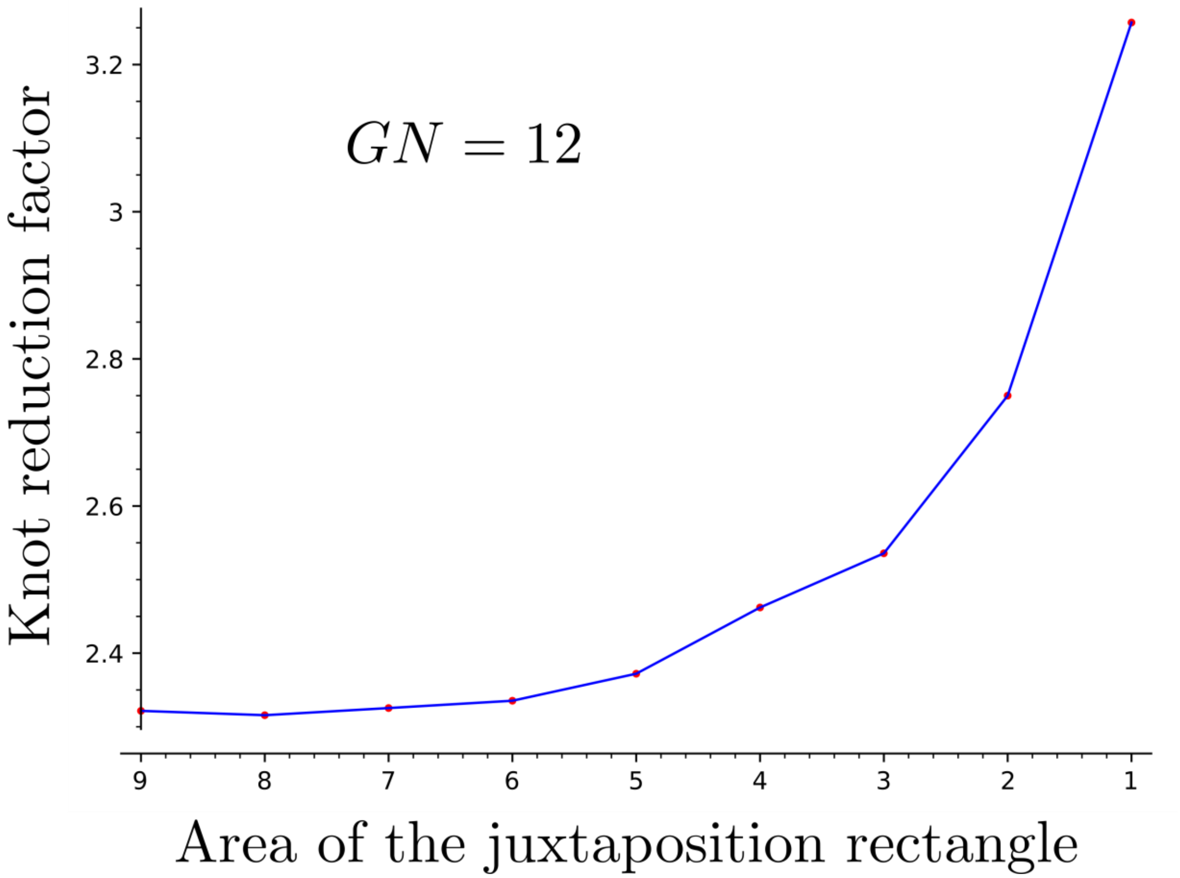

Fig. S5. The knot reduction factor increases with the tightness of hooked juxtapositions. The curve gives the knot reduction factor in *GN* 12, plotted as a function of the rectangle area enclosed by the interleaving strands in hooked juxtaposition. For each value $1 \leq l \leq 9$, the knot reduction factors is computed as the ratio of the number of passages from unknots to trefoils over the number of passages from trefoils to unknots, and the passages happen at hooked juxtapositions with rectangles of size $\leq l$. It is apparent how decreasing such area increases the knot reduction factor.
